# Macro-/Micro-Controlled 3D Lithium-Ion Batteries via Additive Manufacturing and Electric Field Processing

**DOI:** 10.1038/s41598-018-20329-w

**Published:** 2018-01-30

**Authors:** Jie Li, Xinhua Liang, Frank Liou, Jonghyun Park

**Affiliations:** 1 Department of Mechanical and Aerospace Engineering, Missouri University of Science and Technology, Rolla, MO 65409 USA; 2 Department of Chemical and Biochemical Engineering, Missouri University of Science and Technology, Rolla, MO 65409 USA

## Abstract

This paper presents a new concept for making battery electrodes that can simultaneously control macro-/micro-structures and help address current energy storage technology gaps and future energy storage requirements. Modern batteries are fabricated in the form of laminated structures that are composed of randomly mixed constituent materials. This randomness in conventional methods can provide a possibility of developing new breakthrough processing techniques to build well-organized structures that can improve battery performance. In the proposed processing, an electric field (EF) controls the microstructures of manganese-based electrodes, while additive manufacturing controls macro-3D structures and the integration of both scales. The synergistic control of micro-/macro-structures is a novel concept in energy material processing that has considerable potential for providing unprecedented control of electrode structures, thereby enhancing performance. Electrochemical tests have shown that these new electrodes exhibit superior performance in their specific capacity, areal capacity, and life cycle.

## Introduction

Although remarkable advances have been made in lithium ion batteries (LIBs) during the past several decades, higher energy and power densities are still required for portable devices, transportation, and stationary applications^1–3^. Even though gravimetric capacity is one of the most utilized metrics in measuring LIB performance, the amount of materials in an electrode actually determines the energy and power of the LIB. Thus, the requirement for a high tap density is of considerable importance for various applications. The conventional strategy towards a high tap density is to add more material to a higher packing density. While an increase in the volume fraction of an active material improves the transport of lithium ions and electrons on the solid phase, it impedes the transport of lithium ions in an electrolyte. For this reason, increasing packing density is not always desirable. An alternative strategy would be to add more materials by increasing the thickness of the electrodes. This approach, however, limits the transport of ions and electrons, resulting in poor power performance and inefficient utilization of materials. The goal of this paper is to present a means for circumventing these challenges to conventional structures through a new concept for electrode structures, based on macro-micro-controlled three-dimensional (3D) electrodes that can facilitate the transport of the species. An optimized 3D structure permits a facile transport of ions, via a short diffusion path with an enhanced electrochemical reaction, through a higher interface area. (Figure S1). For this reason, 3D structured electrodes are considered to have a huge potential for improving battery performance^3–7^.

Recently, an extrusion-based additive manufacturing process has been proved to provide many advantages compared to other additive manufacturing technologies, such as aerosol jet and ink jet printing. Not only is it inexpensive and flexible enough to fabricate more complex geometry designs, but it can be applied to a wider selection of materials with a high mass loading^8,9^. In particular, the extrusion-based additive manufacturing technique appears to be a very promising method for fabricating 3D battery electrode structures^5,7,10–13^. Unfortunately, the preparation of a proper composition of paste for the extrusion process is rather demanding because of the need to prevent clogging of the nozzles, promote a bond between each filament, and keep a controlled feature geometry after extrusion^14–16^. In addition, for LIB applications, the chemical components in a paste can significantly affect battery performance. For instance, additional binders for improving mechanical integrity would decrease ionic and electronic conductivity.

Another approach that could improve battery performance is to deploy well-organized individual particles in an electrode. Modern batteries are fabricated by casting randomly mixed slurries onto current collectors. These randomly distributed particles (active particles or additive particles) easily agglomerate to form weak spots that can cause a bottleneck in the electrochemical reaction. Also, particles can become an isolated group within the network and, consequently, this isolated group does not perform its essential duty, but hinders the transport of species instead. Further, because a random structure may create a long path for transport, a well-organized structure will provide better responses and superior performance, as compared to a randomly distributed structure. Battery electrodes, with controlled structures at the micro/nano level (such as nanotubes and 3D nanostructures), have been synthesized based on a top-down approach that includes the use of lithography tools, but these are expensive and time-consuming^17–25^. An opposite approach is to fabricate structures, via a bottom-up approach using chemical or physical reactions. In particular, utilizing an electric field (EF) is an effective approach because it is easily implemented and it provides a long-range effect of electrostatic interactions^26–29^. It has been found that an EF could efficiently manipulate particles in a colloidal slurry, including a “chain effect” by moving and rotating particles in a slurry under an external EF.

This paper details a new innovative approach for fabricating 3D structured electrodes, in which three-dimensional features can be simultaneously controlled at the macro-micro-levels (which the conventional manufacturing process cannot do). The proposed process integrates the extrusion-based additive manufacturing process for macro-control and an EF for micro-control. This is a new unique method for manufacturing battery electrodes that has the potential for providing synthetic control of materials architectures, such as particle network, geometries, and integration. This could lead to transformational enhancement of key energy storage parameters that include capacity, energy density, and life cycle.

## Results

### Solids loading impact

**Figure 1:**
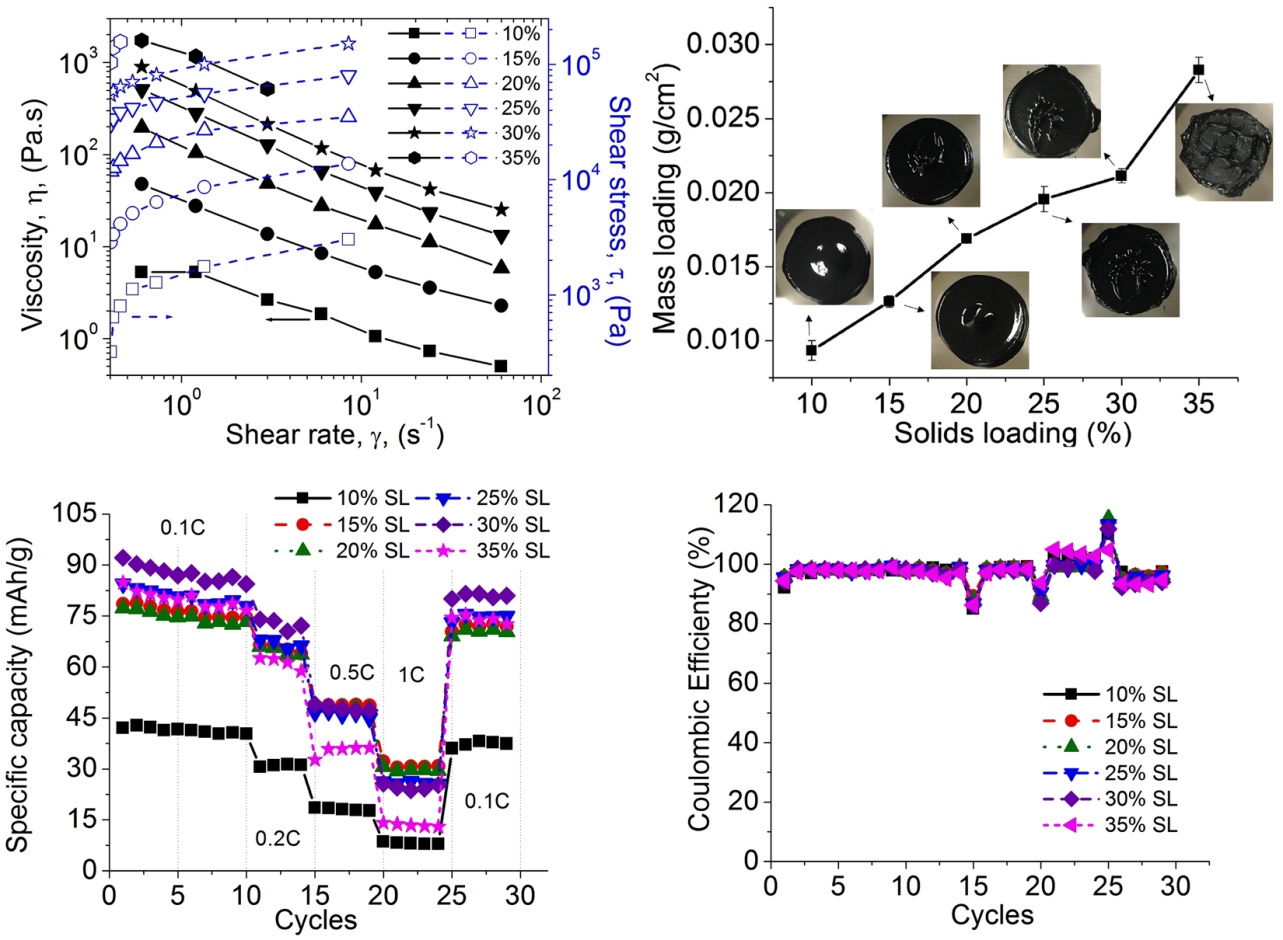
Solids loading (SL) impact on (**a**) paste rheology, (**b**) mass loading, (**c**) specific capacity, and (**d**) coulombic efficiency in a range of 10% SL to 35% SL.

Constituent materials should be organized to promote high conductivity, robust mechanical strength, a high specific area, and superior battery performance. To achieve these, two aspects must be simultaneously considered, including shaping the structure and the corresponding battery functionality. To shape target structures, via an extrusion-based additive manufacturing process, many factors should be considered, including the impact of the electrode’s constituent materials, solids loading (SL) (volumetric ratio of solids in a solution) to prevent clogged nozzles, bonding strength between each filament, and features to be retained after extrusion. These features can all be characterized by two key physical properties of paste, viscosity and shear stress. For battery function, a large amount of active material would contribute to higher capacity, together with appropriate amounts of a conductive material and binder. However, excessive amounts of an additive material could interfere with species transportation. To understand and determine the fundamental requirements for the slurry, first, the effect of SL on battery fabrication and performance were studied based on the conventional structures without any geometric control. Six LiMn_2_O_4_ (LMO) pastes, with different SLs, were prepared from 10% to 35% (in 5% increments, Table S1). Rheology test results (Fig. 1a) indicated that all of the pastes exhibited a shear-thinning behavior, implying that they could be extruded and controlled by the extrusion process. The effect of SL was that the viscosity increased with increasing SL, and the 30% and 35% SL pastes (10^3^ Pa.s and 10^5^ Pa) showed two orders of magnitude higher in viscosity and stress than the 10% SL paste did (10 Pa.s and 10^3^ Pa). This high viscosity was related to the prevention of the collapse of a 3D extruded structure, which will be discussed later.

To determine the relationship between the SL and mass loading (ML) (weight of active materials in a unit electrode foot area), conventional structures with 160 μm electrode thickness were examined. As shown in Fig. 1b, a linear relationship was observed between paste SL and electrode ML. By increasing the SL from 10% to 35%, the ML increased 2.8 times and achieved 0.028 g/cm^2^. For LIB applications, in general, a higher ML was required to increase energy density. When the structure was too dense, however, the transport of lithium ions in the electrolyte was hindered, as described earlier. Therefore, there is an optimal SL due to the trade-off between the transport properties of the solid and electrolyte phases. Figure 1c and d show battery cycling performance with different SLs. A 10% SL produced the lowest mass loading (0.01 g/cm^2^) and a very low specific capacity (40 mAh/g). This poor specific capacity (from the 10% SL) could be caused by poor percolation of the electrode (Figure S2c), while the high SL paste could cast a dense electrode (Figure S2d). As evidenced, Figure S1b shows a high charge transfer resistance (200 Ω) of the 10% SL cast electrode, compared to other high SL cases. The 30% SL showed a maximum specific capacity of 98 mAh/g, while the 35% SL showed a lower capacity of 85 mAh/g. For coulombic efficiency (Fig. 1d), all samples exhibited good performance. A quick change in the coulombic efficiency was observed when the C-rate changed, but it quickly stabilized. This was caused by a residual concentration gradient inside particles caused by the previous cycling. In summary, by considering both paste properties and battery performance, the 30% SL was selected for the fabrication of macro-micro controlled electrodes (to be discussed later).

### Macro-controlled 3D structure

**Figure 2:**
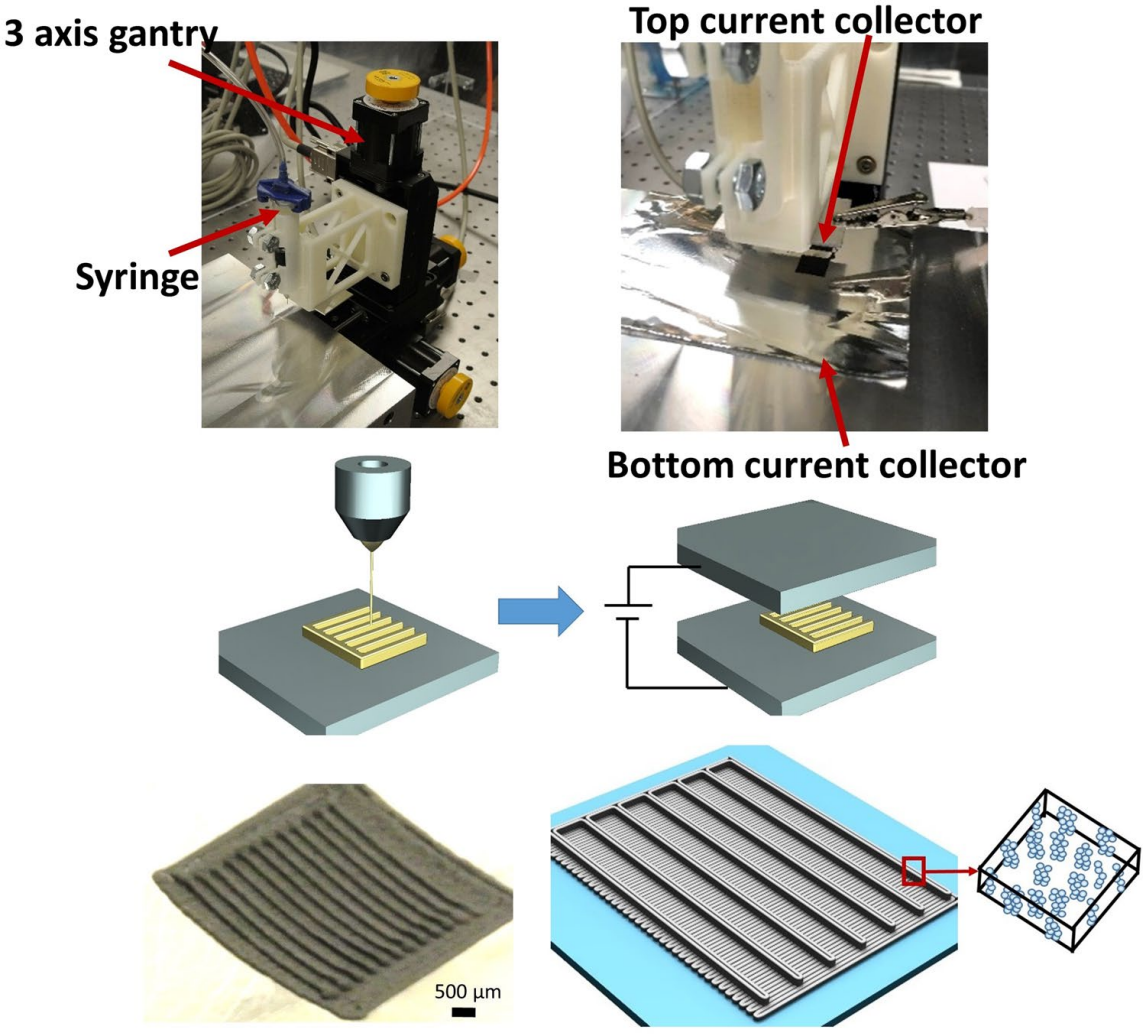
Illustration of (**a** and **c**) additive manufacturing system, (**b** and **d**) electric field treatment process, and (**e** and **f**) macro-micro controlled structure.

**Figure 3:**
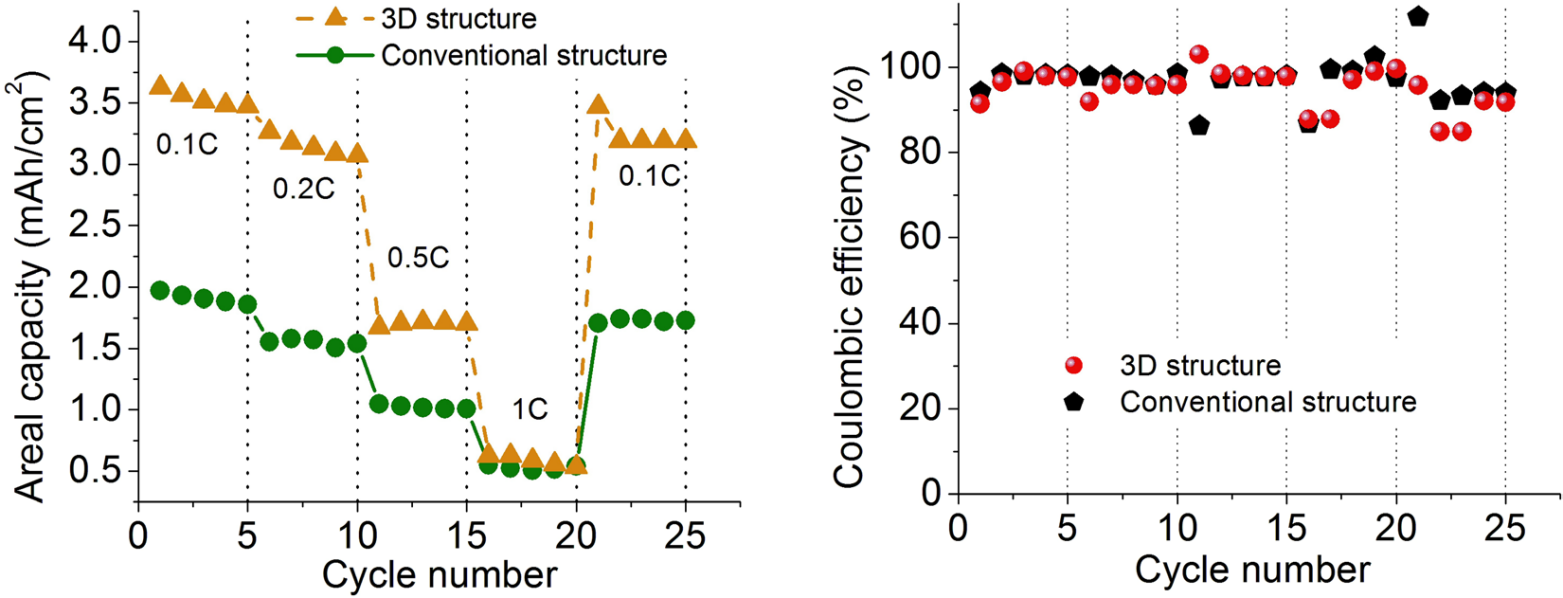
Cycling performance of (**a**) the conventional structure and macro-controlled 3D structure (**a**) areal capacity and (**b**) coulombic efficiency.

The proposed extrusion-based additive manufacturing process is shown in Fig. 2. Figure 2a and c depict the actual system and a schematic diagram, respectively, while Fig. 2b and d show the electric field process (described in the next section). This extrusion process was used to fabricate a macro-controlled 3D structure (a hybrid 3D structure composed of a digital structure on a conventional laminated structure). Figure 2e shows one example of printed 3D electrodes. A macro-controlled hybrid 3D structure was systematically studied in the authors’ previous work^5^. In this paper, a verification test was first conducted to compare the conventional and micro-macro-controlled structures. This macro-controlled 3D structure was fabricated by adding an interdigitated 3D structure on the top of a conventional laminate structure (160 μm), making the total electrode thickness 270 μm. The thickness of the conventional cell used for the comparison was also 270 μm. Then, each cell was cycled at rates of 0.1 C, 0.2 C, 0.5 C, 1 C, and 0.1 C, with five cycles per each C-rate. As shown in Fig. 3, the cycling test showed that the areal capacity of the macro-controlled 3D structure reached 3.1 mAh/cm^2^, which was 1.7 times higher than that of the conventional structure. In addition, as expected, the capacity reduced at high C-rates due to high ohmic resistance. For coulombic efficiency, in general, both structures had a stabilized value, but a small variation in C-rates after the initial formation cycle (shown in Fig. 3b). During the first five cycles, in particular, the structures showed a lower coulombic efficiency. This was related to chemical side reactions during the formation cycle. For instance, like the Solid Electrolyte Interphase (SEI) layer in the anode, a thin film formed on the cathode particles’ surface, called Solid Permeable Interface, SPI layer. In general, this process consumes the active lithium ions and solvents and causes gas evolution that builds up pressure inside the cell, causing significant capacity fade. For this reason, the inside of the battery is not stable and might show a lower coulombic efficiency during the first few cycles. This phenomenon was also observed during the first few cycles in our previous experiments^5^.

**Figure 4:**
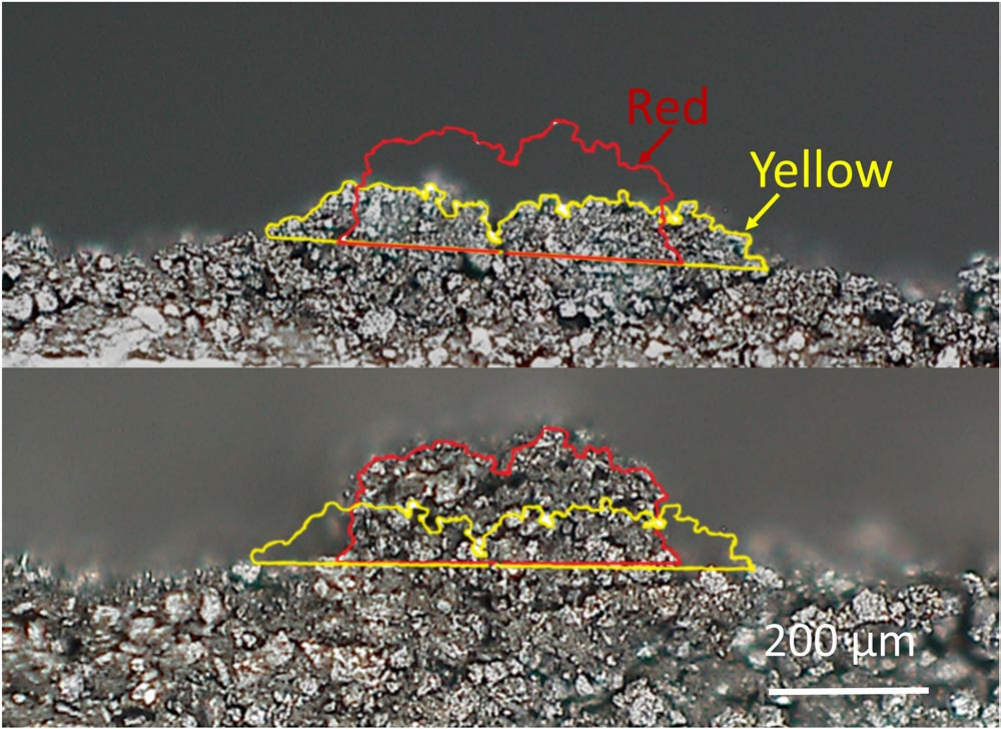
Effect of a hot plate on electrode geometry: (**a**) without a hot plate and (**b**) with a hot plate. Each contour plot represents the boundary of the structure. For comparison, the contour plots are overlapped, as shown in both (**a**) and (**b**) above.

As described earlier, maintaining a 3D structure after extrusion is critical for gaining the benefits of the structure. In this work, to prevent the collapse of an extended 3D structure, a hot plate (HP) was used as an external heating source to accelerate drying. Figure 4 shows cross sections of two electrodes, without (Fig. 4a) and with (Fig. 4b) an HP. As shown in Fig. 4a, the 3D structure without the HP did not keep the desired interdigitated structure but collapsed into an uncontrolled shape. The contour plots, marked on the images, represented the boundaries of each electrode and clearly showed the differences in the final fabricated structures. To contrast them, each line was overlapped in those figures and, when the two electrodes were compared by ImageJ software, a 29% difference was measured between the contour lengths. By considering the same length in the plane, this meant that a 29% reduction in the outer surface area occurred because of the collapse. This 3D structure collapse was related to the drying speed of the paste. When it was naturally dried, without an HP, the collapse happened very slowly in the air (i.e., within approximately 6 to 10 hours), but with an HP, the electrodes could be partially dried and solidified within 1 minute, and dried completely within 10 minutes. Thus, the use of an HP (or another type of heating source) is a good option for accelerating the drying process during fabrication to construct a well-controlled 3D structure.

### Micro-controlled structure

**Figure 5:**
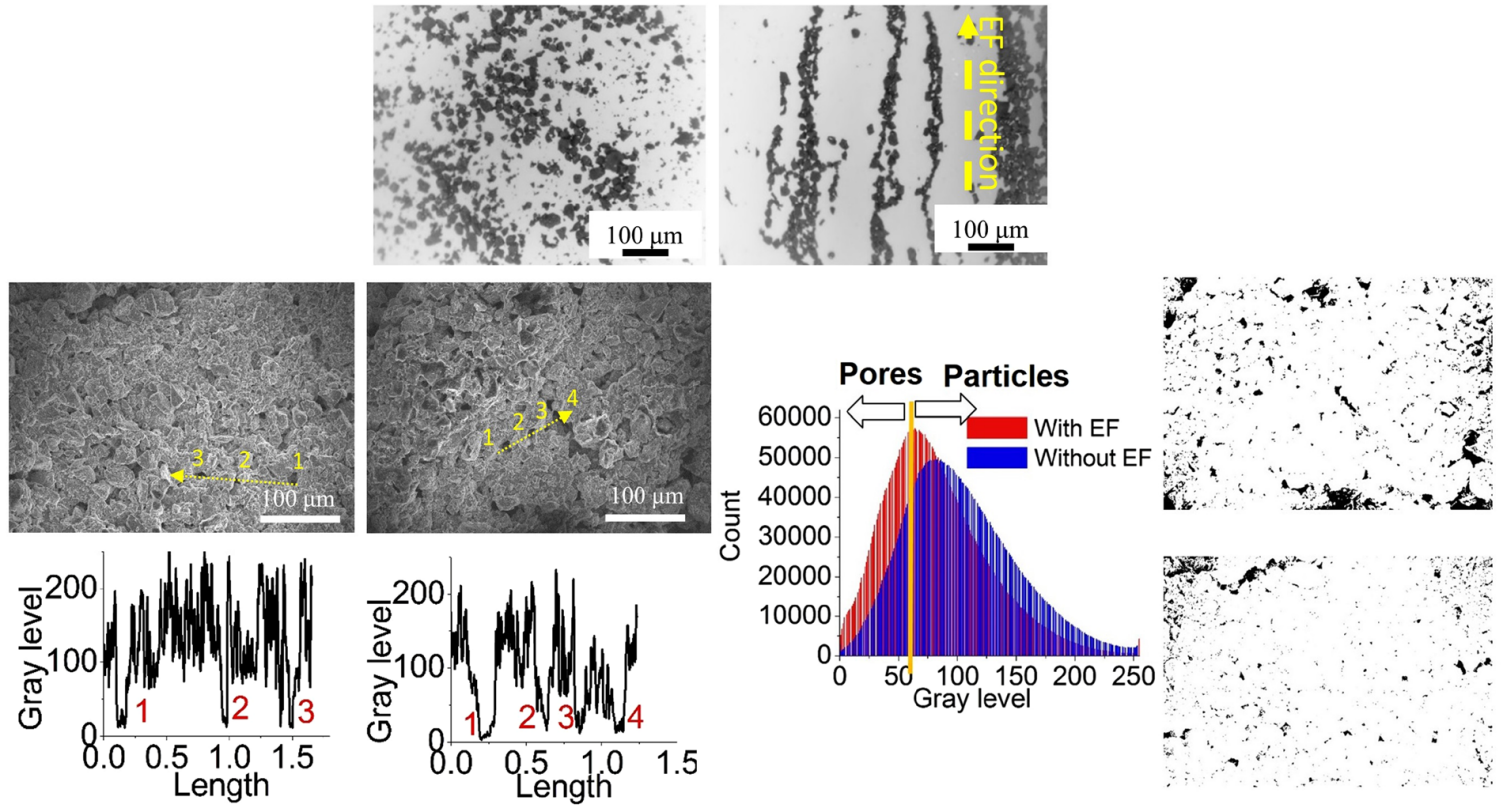
Effect of an electric field on LMO powder (**a**) without EF and (**b**) with EF. SEM images of electrodes (**c**) with EF and (**d**) without EF, Gray level profiles (**e**,**f**) of the lines shown in the SEM images, Gray level distribution (**g**) of two samples, and Pore distribution after an adjustment of color threshold for the electrodes (**h**) with EF and (**i**) without EF.

As described earlier, a well-ordered internal structure is another way to enhance battery performance. The remaining question, concerning materials processing, is how to fabricate an electrode with a controlled internal structure. In this work, an EF was used. Figure 2b and d show the setup used for processing by applying an EF to create organized nanostructures in an electrorheological fluid, composed of particles dispersed in a slurry. The dispersed particles tend to line up and form a chain parallel to the applied EF. Such behavior can be attributed to electric polarization interaction, a pairwise dipolar interaction between particles. Particles, with the same polarization direction, will repel each other if they remain on a plane that is perpendicular to the EF, but the interaction becomes attractive when the two particles shift and are relative to each other by one radius^26–29^. Figure 5a and b show a simple demonstration of this chaining process for battery materials. A slurry of LMO particles in an N-methyl-2-pyrrolidone solvent (NMP) was cast on a glass substrate, and then an EF was applied, along the vertical direction (as shown in the figure). The images were first captured by a stereo microscope (Amscope Inc.) while the particles were randomly distributed on a glass substrate without an applied EF (Fig. 5a). Then, when an EF was applied, the particles moved toward the current collector and, finally, rearranged as “chains” (Figs 5b and S3).

Although these results confirmed the responsive behavior of battery materials to an EF, the viscosity of slurry for practical battery electrodes was high. One important question that needed to be answered concerned the mobility of particles in high viscosity slurry. To answer this question, conventional structure electrodes were fabricated with and without an EF. To compare these electrodes, the Brunauer–Emmett–Teller (BET) test was administered for surface area, Scanning Electron Microscopy (SEM) analysis was conducted for surface morphology, and X-Ray Diffraction (XRD) measurements were made for microstructure orientation. The surface area of particles in electrodes is one of the critical factors that determine battery performance. From the BET test, it was found that the surface area of the electrode under an EF was found to be about twice that of the electrode without EF. The measured surface areas were 3.5 m^2^/g and 1.7 m^2^/g, respectively. Next, to visualize the difference, SEM images with different orientations were taken. As shown in Figs 5c,d, S4 and S5, it was difficult to distinguish them by appearance alone. For further analysis, a graphical interpretation was made by using ImageJ software (a common approach for porosity analysis)^30^. For this, the images were cropped to remove label bars, and adjusted to maximum and minimum brightness and contrast. To identify the pores, a threshold value of 70 gray level was selected by observing the sharp drop in line profiles, which represented the pore boundary (Fig. 5c–f). This value was confirmed with a dozen of pores and then used to measure the areal porosity of the whole binary images (Fig. 5g). This analysis showed that the electrode with an EF had more porosity (8.5%) than the electrode without EF (3.9%), as illustrated in Fig. 5h and i. This result was consistent with the conclusion obtained from the BET test.

Another interesting piece of evidence, concerning the responsive behavior of battery materials to an EF, was obtained from an XRD measurement. The XRD test was performed for electrodes without EF and with EF (at different applied voltages) at different drying times. A maximum applied voltage (10 kV) was selected that would avoid sparks during the process that could cause damage to the electrodes. For comparison, half of the maximum voltage (5 kV) was also applied. Additionally, based on a hypothesis that drying time will affect the microstructure, different drying times (3 h and 6 h) were compared. When the applied EF was turned off before the electrode was fully dried, a Brownian motion might break the formed structures because of the EF effect. Based on a rough estimation, the electrodes were partially dried in 3 hours and fully-dried in 6 hours. On the other hand, when an HP was used, the drying time was 1 minute for half drying and 10 minutes for full drying.

**Figure 6:**
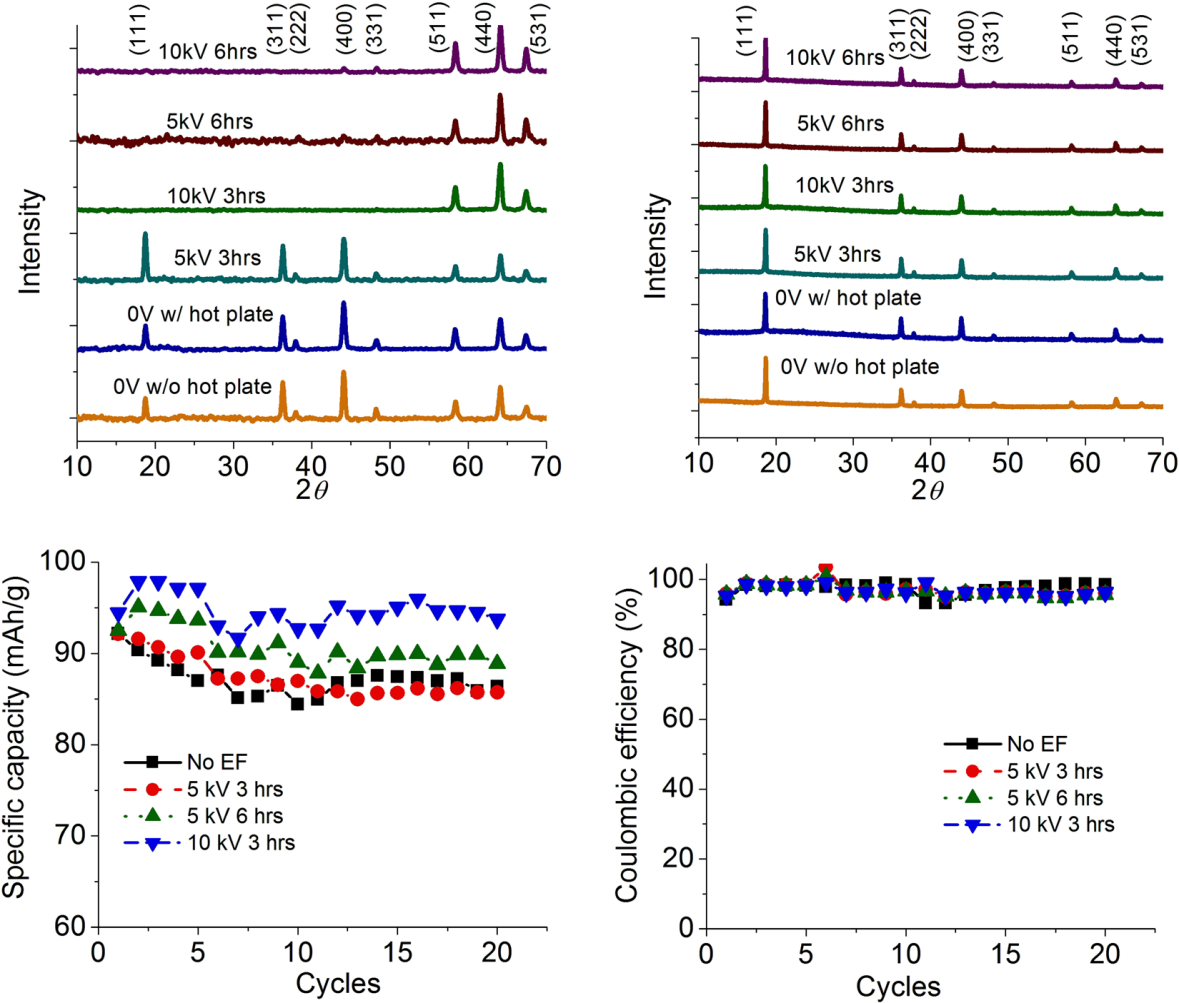
XRD results obtained from electrodes under different conditions: (**a**) electrode film samples and (**b**) electrode power samples, and (**c**) and (**d**) cycling performance with different conditions.

For XRD measurement, each sample was tested based on two forms, including a form of the whole electrode itself and as the powder after breaking the same electrode. Samples with 0 V without an HP were used as the control group. The peaks corresponding to the control group (shown in Fig. 6a) are well matched with the reported values in the literature^31,32^. At a low voltage (5 kV) with a short drying time (3 h) and 0 V with HP samples, the peaks were the same as the control sample. However, the peaks in <111>, <311> and<222> were missing for long periods of time from electrode samples with high applied voltages. A possible reason for this was a preferred orientation of the particles under the applied EF. In order to confirm this, the measured electrodes were broken into a powder and measured by XRD again. For all samples, the missing peaks showed up again (Fig. 6b), indicating that there was no longer any preferred orientation. These series of XRD measurements proved that when the applied EF was too small, or the drying time was too short, the EF effect could not be expected. This supported the proposed reason for the missed peaks above.

Next, four samples with conventional structures (no EF, 5 kV/3 h, 5 kV/6 h, and 10 kV/3 h) were assembled into half-cells to examine the effect of EF on battery performance. As shown in Fig. 6c and d, the electrodes with 10 kV/3 h and 5 kV/6 h showed higher capacities than those of the electrodes without an EF and with 5 kV/3 h. Hence, the samples effectively-treated by an EF (Fig. 6c) showed improved results. For the coulombic efficiency (Fig. 6d), all of the samples showed similar values of around 95% to100%.

### Macro-micro controlled structure

**Figure 7:**
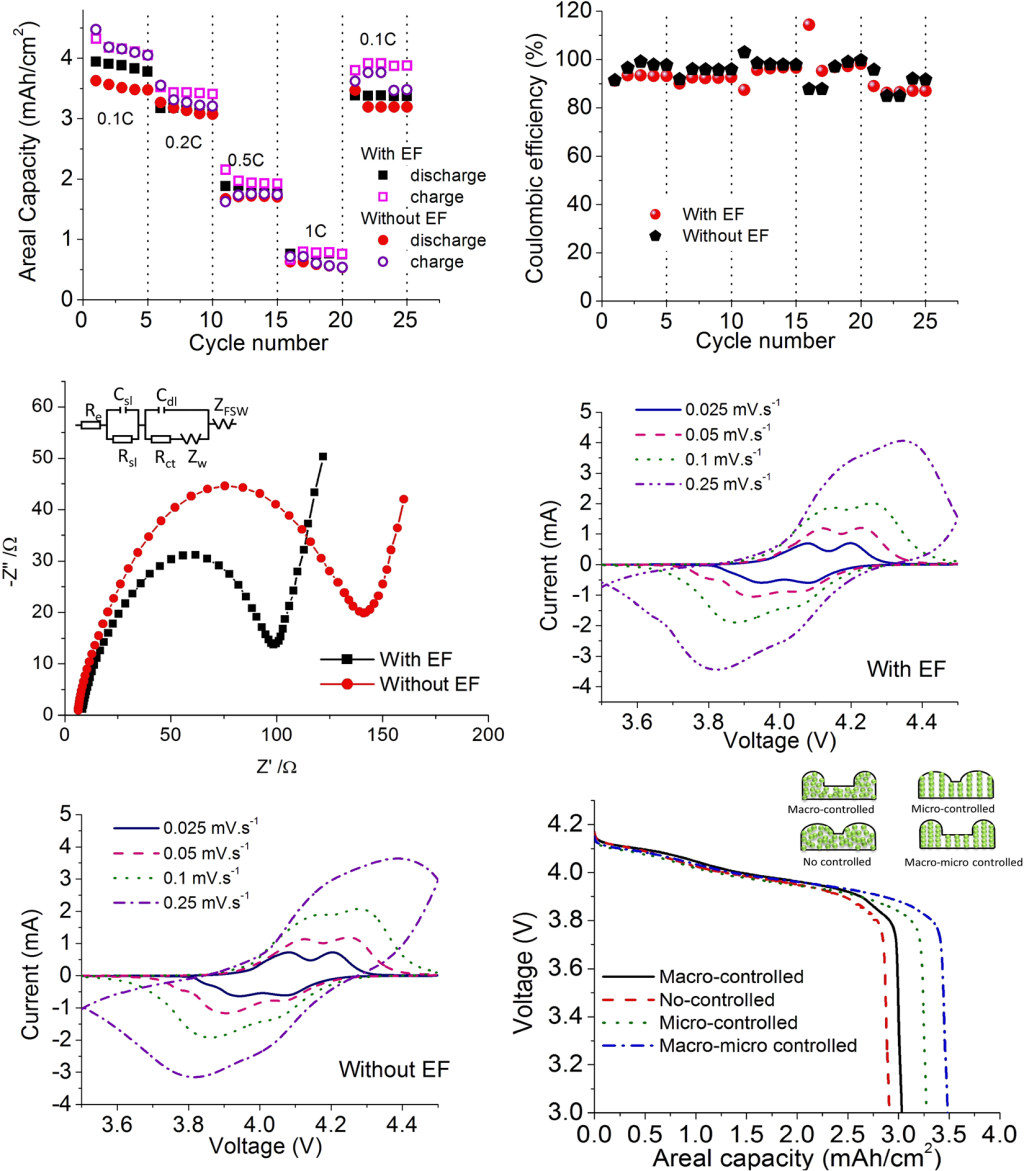
Comparisons of the performance of structures with and without EF of (**a**) cycling with 0.1 C, 0.2 C, 0.5 C, 1 C, and 0.1 C; (**b**) coulombic efficiency; (**c**) impedance and cyclic voltammetry; (**d**) with EF; and (**e**) without EF; (**f**) voltage profile comparison of the four configurations.

As the final goal, both macrostructure (additive manufacturing process) and microstructure (electric field process) were controlled simultaneously. For the macrostructure control, a 270 μm hybrid 3D electrode, which showed the best performance in the previous section, was constructed. Next, an EF with 10 kV was applied to those hybrid electrodes. For comparison, some of the hybrid electrodes were not applied with an EF. Then, the cells were cycled at different C-rates (0.1 C, 0.2 C, 0.5 C, 1 C, and 0.1 C), with five cycles per each C-rate (shown in Fig. 7a). All samples showed stable performance, along with slight decreases in capacity. As expected, the capacity was reduced at high C-rates due to high ohmic resistance. However, the sample with an EF showed greater areal capacity than the sample without an EF did, even when C-rates were high. For instance, the areal capacity of the sample with an EF (0.76 mAh.cm^−2^) was about 1.21 times that of the sample without EF (0.63 mAh.cm^−2^) at 1 C. After returning to the low 0.1 C, both cells showed stable performances, but the one with an EF (3.38 mAh cm^−2^) showed 7% more areal capacity than the one without EF (3.18 mAh cm^−2)^. The columbic efficiency of both samples (with and without EF) was stabilized after a small drop between different C-rates, but they showed similar values, as shown in Fig. 7b.

Nyquist plots were made for both samples (Fig. 7c). The original data were fitted with a circuit diagram model of R(CR)(CRW)W^33^. The high-frequency intercept at the *Z*′ axis corresponded to the ohmic resistance, *R*_*e*_ (which represented the resistance of the electrolyte), and the semicircle in the middle-frequency range indicated the charge transfer resistance, *R*_ct_^34^. The Warburg impedance, *Z*_w_, was related to a combined effect of the diffusion of lithium ions on the electrode/electrolyte interfaces, which corresponded to the straight sloping line at the low-frequency end^35^. It can be seen that both cells had a similar (7 Ω) ohmic resistance, but the semicircle of the EF-processed 3D structure was smaller than that of the 3D structure without an EF. From the fitted impedance parameters, the charge transfer resistance, *R*_ct_, of the 3D printed and EF processed 3D structure (*R*_ct_ ≈ 100 Ω), was smaller than that of the 3D printed without the EF (*R*_ct_ ≈ 150 Ω). This indicated that the (de)intercalation process for the EF-processed 3D structure was easier than that for the sample without the EF^35–37^. This indicated that the EF-processed electrode greatly enhanced the transport of lithium ions.

The cyclic voltammetry curves with EF (Fig. 7d) and without EF (Fig. 7e) indicated that both of the samples had the same polarization values of around 3.8, 4.1, and 4.2 V, which was reasonable because the EF process did not affect the chemical properties (Fig. 6b). In addition, when the two different scan rates (from 0.025 to 0.25 mVs^−1^) were compared, both samples had similar shapes when the scan rate was lower than 0.25 mVs^−1^. The sample with an EF showed a more symmetrical shape at a high scan rate, which meant that the sample with an EF had better rate capability than the sample without EF did.

As shown in Fig. 7f, the voltage profile of four different configurations of the 3D printed batteries with the same thickness of 270 μm were compared; they included (1) no controlled structure (i.e., without HP and EF); (2) macro-controlled structure (i.e., without EF); (3) micro-controlled structure (i.e., without HP); and (4) macro-micro controlled structure (i.e., with HP and EF). As discussed earlier, the process using an HP improved the external 3D structure morphology, while the EF process increased the particle order inside the electrode. The sample with an HP provided 30% more surface area than the sample without HP did and, similarly, the applied EF doubled the surface area of the electrode. For the battery responses for those cells, first, four samples showed a very similar voltage drop, indicating that the ohmic drop was not significantly affected by the fabrication process. Next, as compared to the conventional structure (1.8 mAh/cm^2^), the capacity increased to 2.8 mAh/cm^2^ with additive manufacturing (no controlled structure). However, this capacity was lower than that of the macro-controlled structure with 3.1 mAh/cm^2^. By micro-controlling, the capacity increased farther to 3.3 mAh/cm^2^, indicating that micro-control had more impact than macro-control did. Finally, the macro-micro controlled structure showed the best performance (3.5 mAh/cm^2^) by simultaneously utilizing the advantages of a 3D structure and electronically ordered particles.

## Discussion and Conclusions

**Figure 8:**
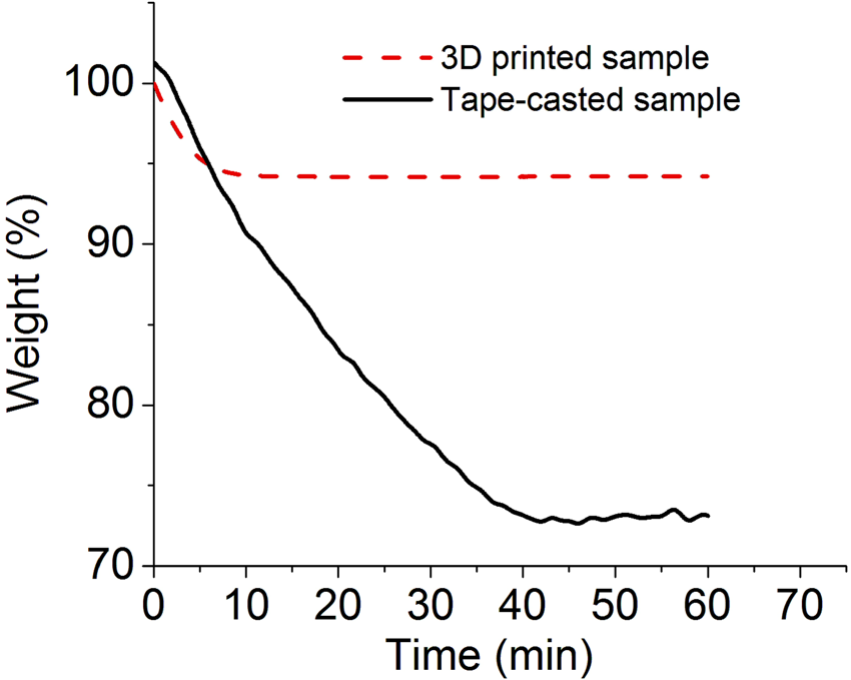
Thermal gravity tests of the 3D printed sample and conventional cast sample.

A well-known disadvantage of the additive manufacturing process is that the three-dimensional printing process takes a long time to mass-produce filaments printed in the x-y direction, and then to print layer-by-layer in the z-direction. Thus, compared to slurry casting (casting any thickness at one time), the time required for printing the structure will increase with increased cell area, thickness, and structure resolution. For example, from our experimental setup (without applying an electric field), a 270 μm thickness, with a 1 × 1 cm^2^ area on a laboratory scale, took 5 minutes for casting, while the extrusion-based printing required about 10 minutes with a 200 μm nozzle size. On the other hand, post-processing was more important and time-consuming from the point of view of the entire manufacturing processing time. Typical casting electrodes required a long drying time in an oven (usually overnight), at 100–130 °C. In contrast, our printing system that contained a heated substrate of 120 °C allowed the samples to be dried quickly within 10 minutes. To compare the two processes, thermogravimetric analysis was performed at 120 °C with a small piece of the 3D sample (50 mg) and a cast sample (30 mg). As shown in Fig. 8, the cast sample took about 40 minutes to dry, while the printed sample did not lose weight during the measurement. This clearly demonstrated that our proposed additive manufacturing method can save more time during the post-processing phase than the conventional method can.

This study proposes a novel multi-scale process to fabricate 3D structure electrodes via combining additive manufacturing and an electric field process. The influence of the processing parameters on a particle network has been clarified in order to improve the quality of electrode structures. The effects of solids loading on paste properties and battery performance were carefully studied initially. Higher SLs increased the paste viscosity and stress that were needed to control the macro-shape after extrusion via extrusion-based additive manufacturing, and to increase mass loading. Based on these two sets of experiments, the 30% SL paste was found to be the optimal loading with high viscosity (10^3^ Pa.s) and high specific capacity (92 mAh/g). The macro-controlled 3D structure showed that the hybrid 3D structure could attain a high areal capacity (3.1 mAh/cm^2^) and double the areal capacity (as compared to a conventional laminated structure).

Further, the effect of using a heating source during printing was studied. It was found that the heating source accelerated solidification of a printed electrode and then helped to retain its shape before solidification was complete. It was also observed that the heating source did not affect the chemical in the electrode materials, and the well-controlled shape was able to improve battery performance (approximately 60%) by increasing the surface area of the electrode, as compared to the conventional structure (Fig. 3a).

For the microstructure aspect, the applied EF ordered the internal structure of the electrode through a “chain effect” that manipulated particles, so that the surface of the electrode increased approximately 200%. The effect of the applied EF was also systematically studied with different EFs and duration times. It was observed that the maximum EF (10 kV) with 3 hours, or a medium EF (5 kV) with 6 hours, would cause the particles to be in a preferred orientation order. Electrochemical performance tests of the samples showed that a higher EF with rapid drying would improve battery performance by approximately 7% (Fig. 6).

Finally, a macro-micro-controlled 3D structure was fabricated, and comparisons were made for battery performances between 3D structure samples with no EF and a maximum EF with an HP. The results indicated that the performance of a macro-controlled 3D structure could be further improved via manipulating the microstructures. Further characterization, including impedance and cyclic voltammetry tests, indicated that the sample with an EF enhanced the transport of lithium ions and had a better capability rate than the sample without an EF did. Voltage profiles from the four different fabrication conditions showed that the macro-micro-controlled structure showed 21%, 16%, and 7% more areal capacity than a structure with no control, a macro-controlled structure, and a micro-controlled structure, respectively (Fig. 7f). The proposed control of extruded structures, with a well-organized distribution of energy materials, demonstrated more superior properties and advantages than structures with randomly distributed materials.

## Methods

### Material Preparation

A LiMn_2_O_4_ (LMO) paste was used to fabricate the electrodes. The paste was prepared by first mixing 85.5 wt% of LMO powder (MTI, 13 μm) with 6.5 wt% of carbon black (CB, Alfa Aesar) and 8 wt% of Polyvinylidene fluoride (PVDF, Sigma-Aldrich). This was then dispersed in the N-methyl-2-pyrrolidone solvent (NMP, Sigma-Aldrich) for different solids loading. The paste was mixed with a SpeedMixer (FlackTeck Inc) at 2000 RMP for 20 minutes at room temperature.

### Electrode Fabrication

An extrusion-based additive manufacturing system was used to extrude the paste into a 3D structure. An aluminum foil piece was fixed on a substrate, prior to printing, which was then used as a current collector after assembly. The extrusion-based additive manufacturing system was a home-built system consisting of a motion subsystem, a hot plate, extrusion devices, and power supply for the EF. The paste was loaded into a 10 ml plastic syringe with a 150 μm nozzle (EFD Inc), and extruded with 80 psi extrusion pressure onto the substrate that moved along the XY-axes. First, a base layer was printed to cover the current collector as a conventional laminated structure. Next, a digital structure was printed on top of the base layer to increase the specific surface area. After printing, a voltage of 10 kV, which was the maximum voltage without a spark, was applied at a distance of 1.25 cm for 10 minutes. A hot plate (at 120 °C) was used to remove solutions quickly. The conventional structures were cast by using a doctor blade on an aluminum foil, followed by EF processing. EFs were applied at a distance of 1.25 cm from the top of the electrode with variable applied voltages and different duration times.

### Materials characterization

The morphologies of an EF-treated sample were characterized with a Scanning Electron Microscopy (SEM, Hitachi S4700) by using secondary electrons at 15 kV accelerating voltage. The SEM images were cropped to remove label bars and were adjusted to the same brightness and contrast by using ImageJ software. A threshold value of the gray level, representing the boundary of pores, was found to be 70 by observing a dozen of pores. This value was used for areal porosity measurements. The X-ray diffraction (Philips X-Pert Diffractometer) test was used to detect the particle orientation caused by the applied EFs. The whole electrode samples and their broken powder samples were compared through XRD tests.

### Assembly

A CR2032 coin cell (Wellcos Corp) was used to assemble a battery in an argon-filled glove box (Mbraun). LMO was used as a cathode, Li foil as an anode, and commercial PP/PE/PP membrane (Celgard) as a separator; the battery was filled with liquid electrolyte 1 M LiFP_6_ EC:DMC 1:1 (Sigma-Aldrich).

### Electrochemical Measurements

The electrochemical behavior of the assembled coin cells was measured from 3 V to 4.2 V by using a battery testing station (IVIUMnSTAT, Ivium Tech). The specific capacity and areal capacity were measured under a 0.1 C-rate, and then the cycling performances were conducted with 0.1 C, 0.2 C, 0.5 C, 1 C, and back to 0.1 C for five cycles. Battery impedance was measured via an electrochemical impedance spectroscopy (EIS) at 3.5 V open circle voltage, and the cyclic voltammetry curves were measured at 0.025–0.25 mV+s^−1^.

## Electronic supplementary material

Supporting Materials
