# Supplementary material for: Macro-/Micro-Controlled 3D Lithium-Ion Batteries via Additive Manufacturing and Electric Field Processing
Source: Sci Rep. 2018 Jan 30;8:1846. doi: 10.1038/s41598-018-20329-w (PMC5789829; doi:10.1038/s41598-018-20329-w)
Supplement: Supplementary file 1 — Supporting Materials [file 41598_2018_20329_MOESM1_ESM.doc]

**Macro-/Micro-Controlled 3D Lithium-Ion Batteries via Additive Manufacturing and Electric Field Processing**

Jie Li^1^, Xinhua Liang^2^, Frank Liou^1^, and Jonghyun Park^1^,*

^1^Department of Mechanical and Aerospace Engineering, Missouri University of Science and Technology, Rolla, MO 65409, USA

^2^Department of Chemical and Biochemical Engineering, Missouri University of Science and Technology, Rolla, MO 65409, USA

Our fundamental scientific reasoning for using the proposed approach is based on the concept that creation of a functional 3D-structured electrode will significantly enhance current battery performance. The considerable demands of diverse applications will be satisfied by using this unique approach that includes a short diffusion path and a high specific area. Along with an understanding of the nature of the “building blocks” of this approach, another important factor for determining product properties is knowing how to “stack” or “arrange” the blocks. The key proposed idea is related to a 3D structured electrode. Our primary reason for expecting better performance by using a 3D structure is because the diffusion path is reduced along with the increased interfacial area. Figure S1 shows the distances between anode particles and cathode particles for a 3D electrode and a conventional electrode. The distance for the 3D electrode is 7.9% shorter than that of the conventional structure (when a 6x6 particles matrix is considered). The shorter distance via the 3D structure means a faster response. The second proposed idea is related to the organization of the building blocks. For a random structure, there may be a chance that some particles (active particles or additive particles) might form an isolated group within the network. Should this be the case, the isolated group will not be able to perform as required but, instead, it will hinder the transport of species. Also, such a random structure could create a long path for transport. Consequently, particles that are organized into a well-developed network should help improve product properties.

**
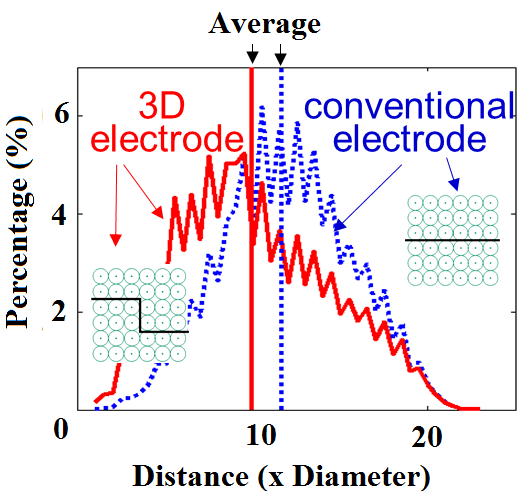
**

Figure S1. Particle distance distribution from anode to cathode for conventional structure and 3D structure.


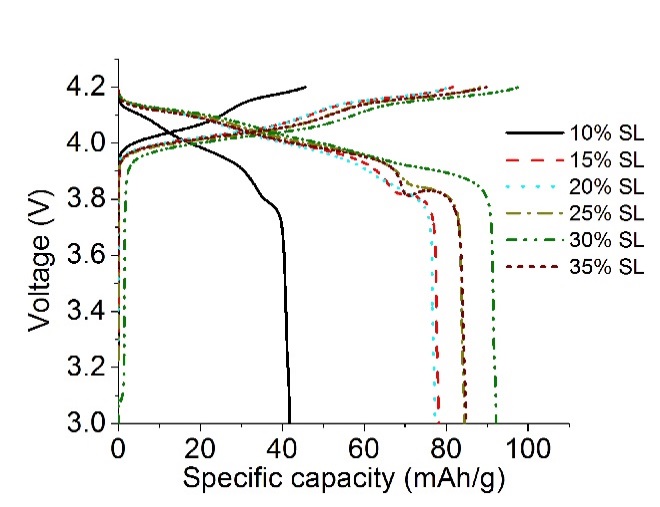

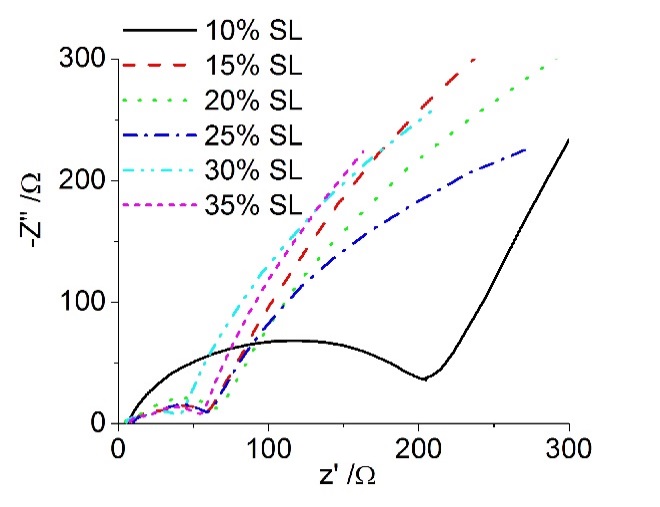
**
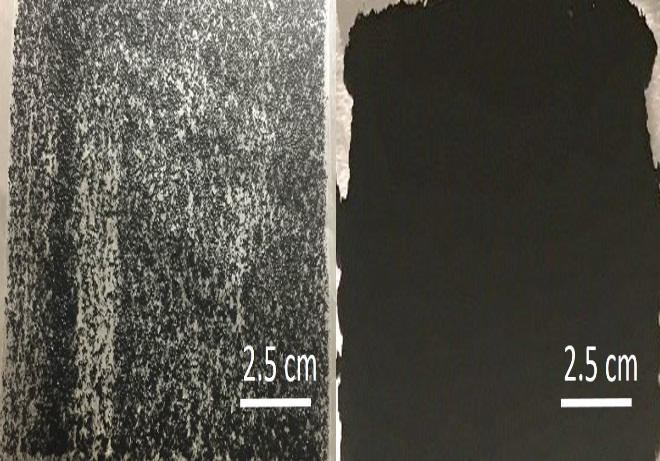
**

(a)

(b)

(c)

(d)

Figure S2. Effect of solids loading on (a) voltage profile, (b) impedance, and (c) poorly connected electrode materials (black) cast on Al foil (white) with 10% SL paste and (d) well cast electrode with 30% SL paste.


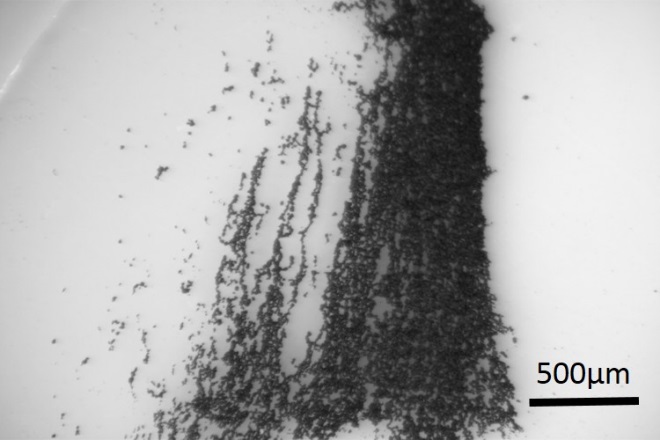

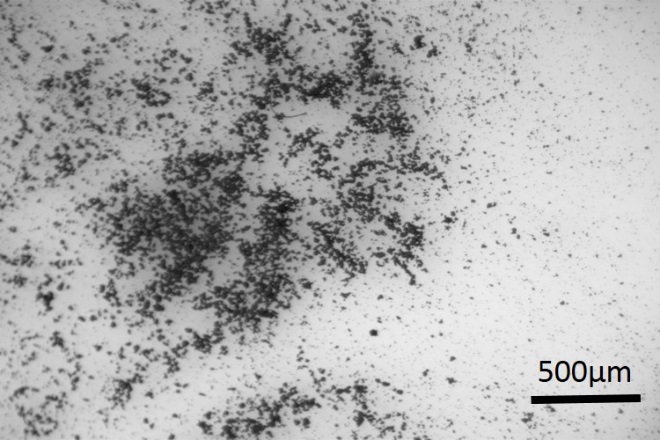


(a)

(b)

Figure S3. Effect of electric field (EF) on LMO powder (a) with EF and (b) without EF

Table S1. Images of pastes with different solids loadings

| 10% | 15% |
| --- | --- |
| 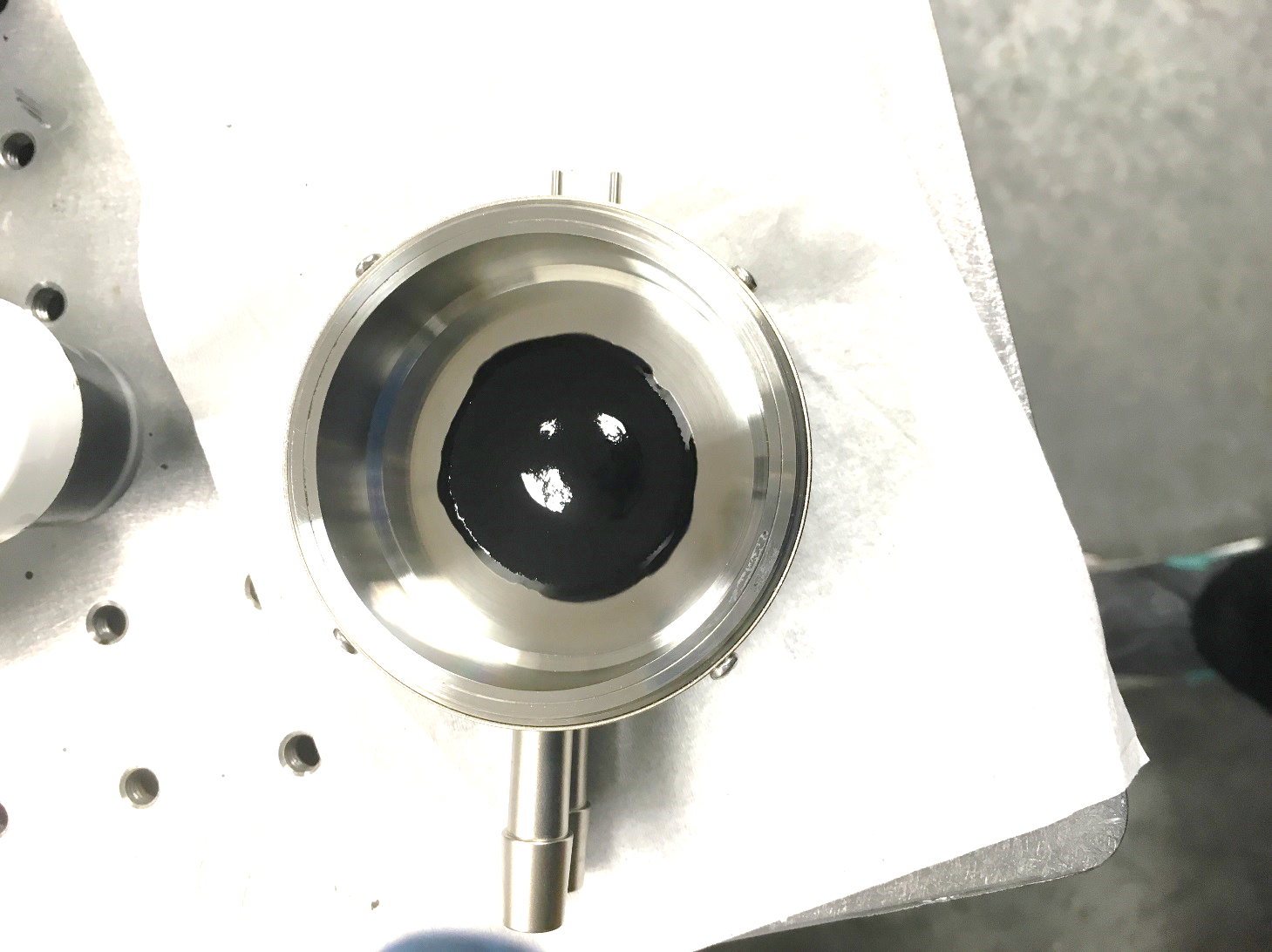 | 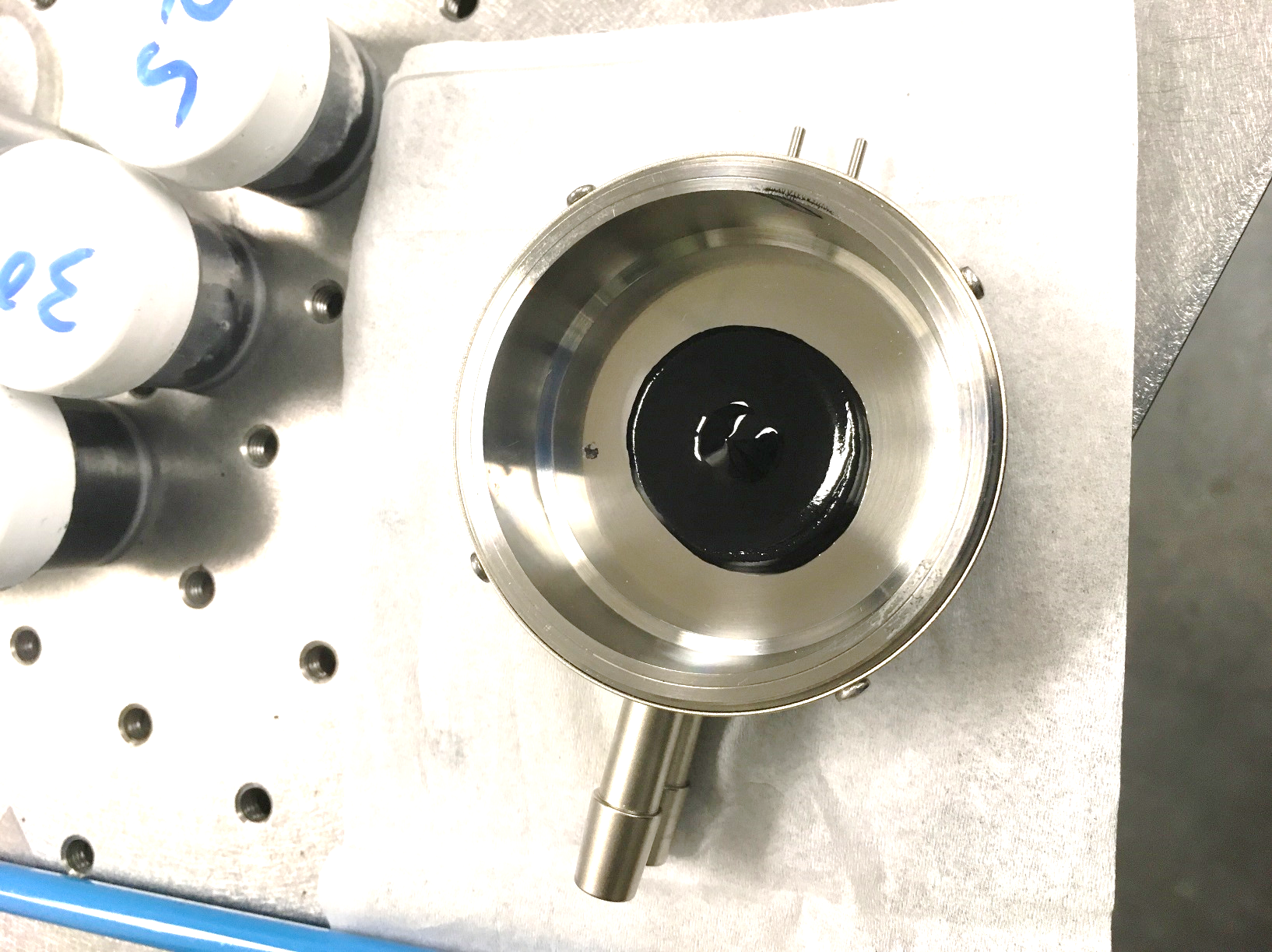 |
| 20% | 25% |
| 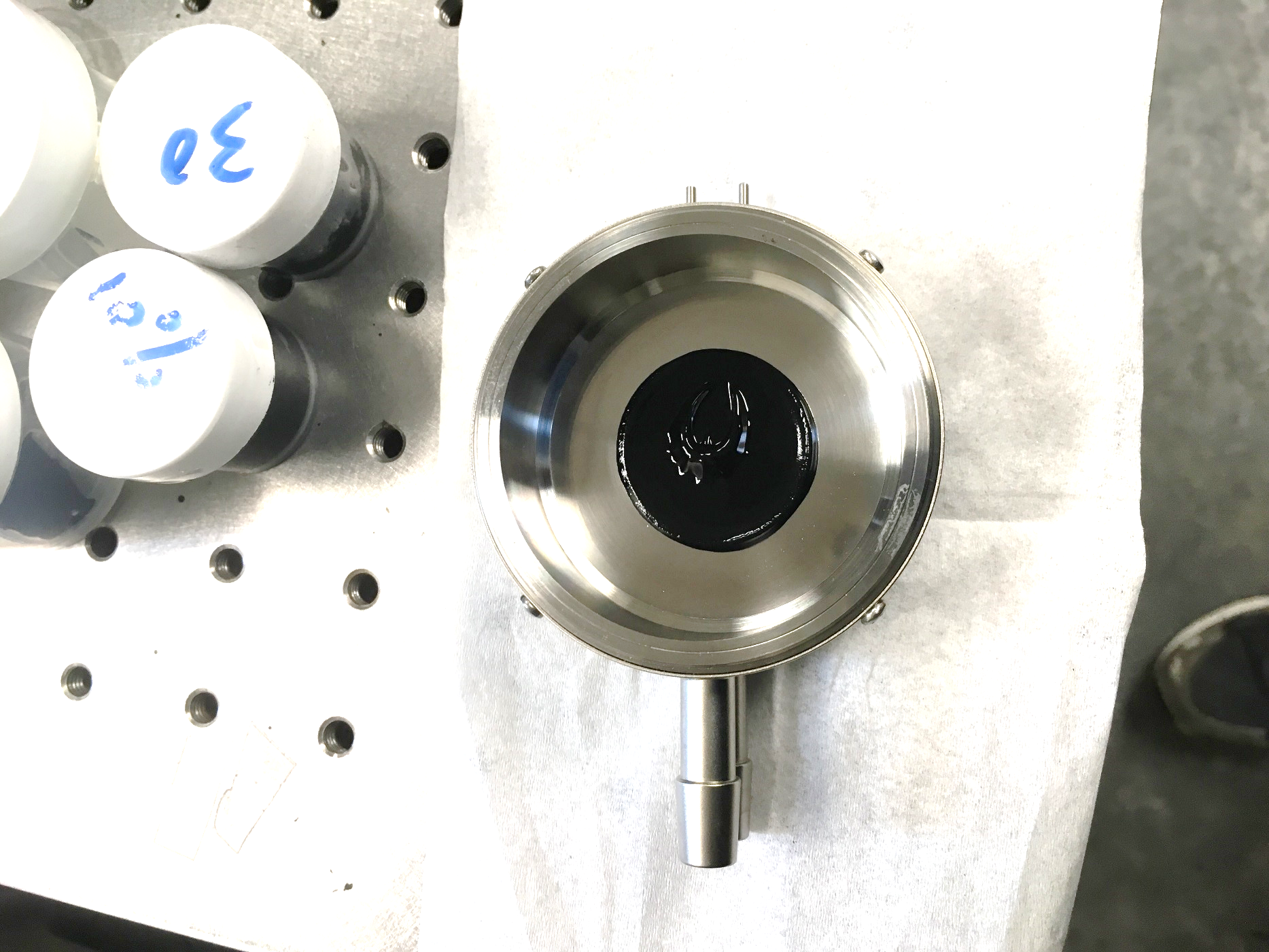 | 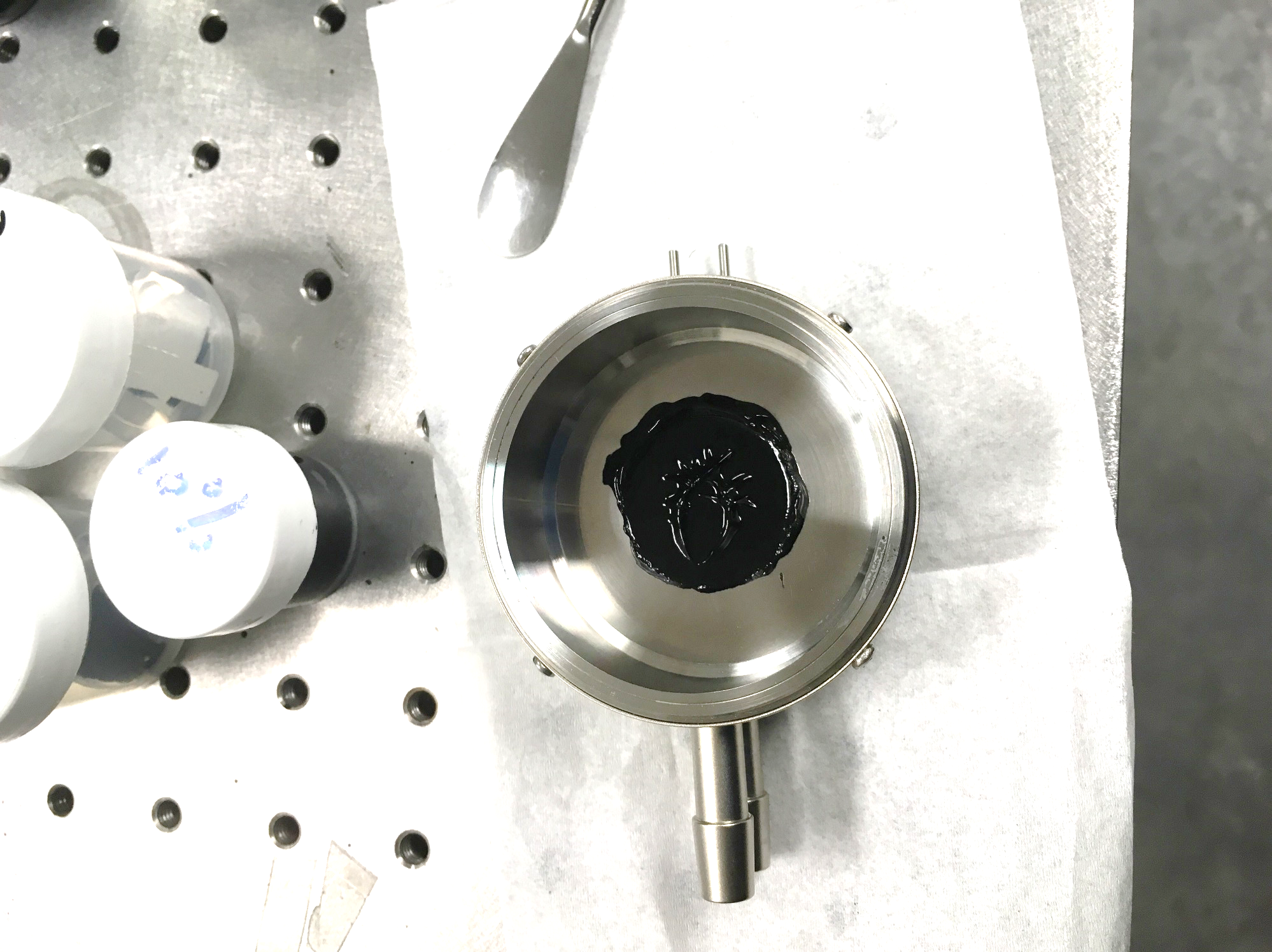 |
| 30% | 35% |
| 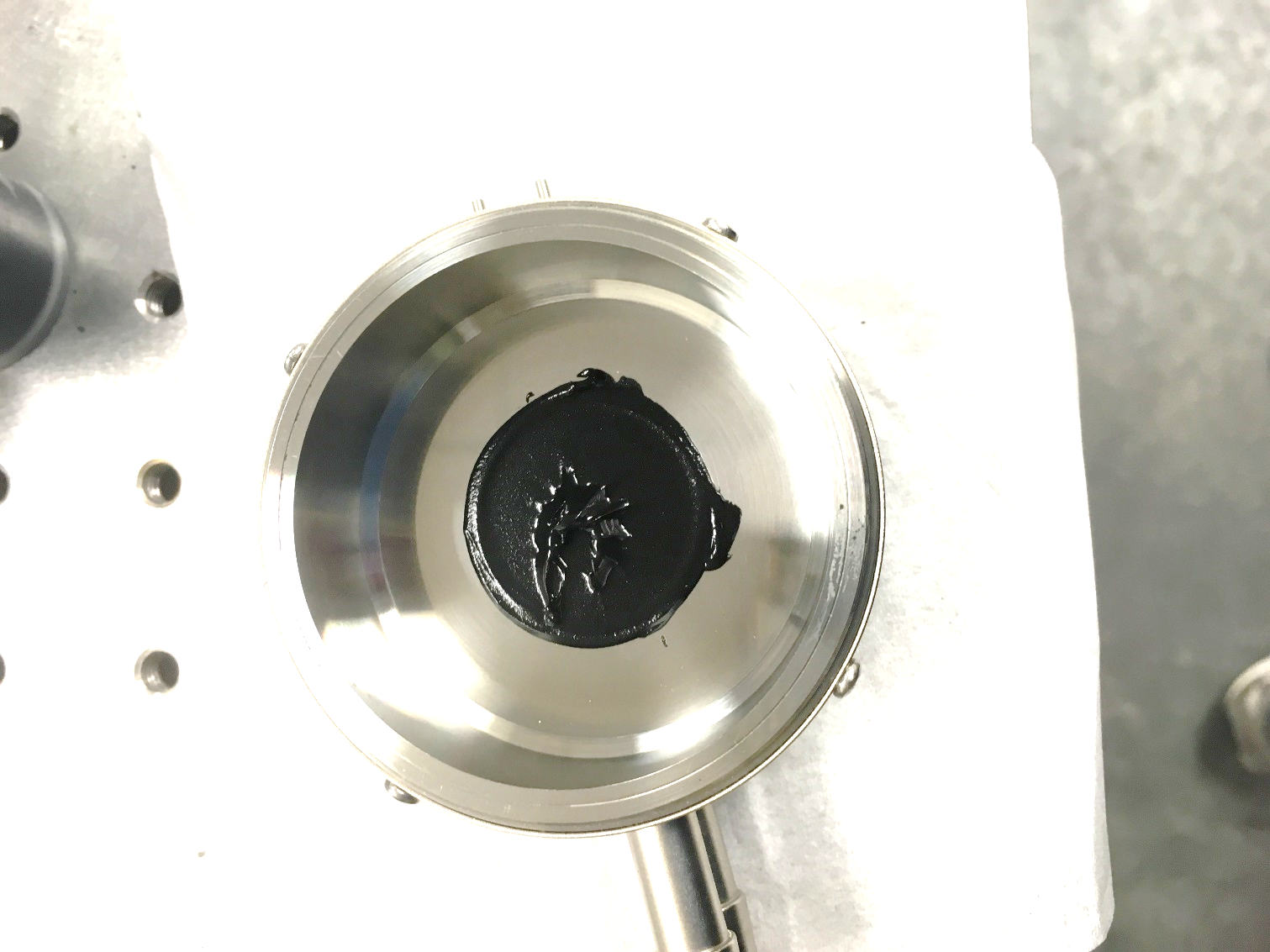 | 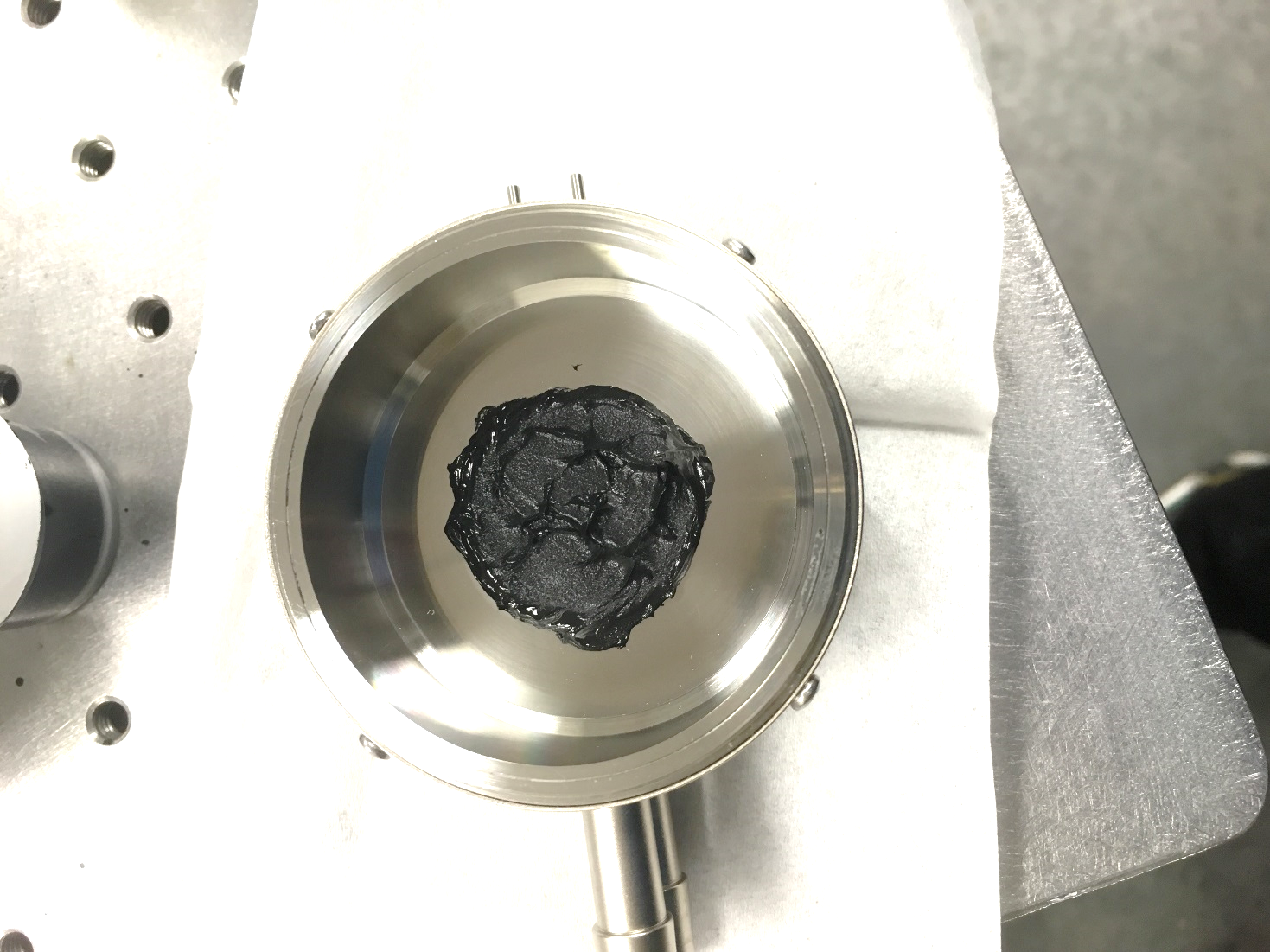 |


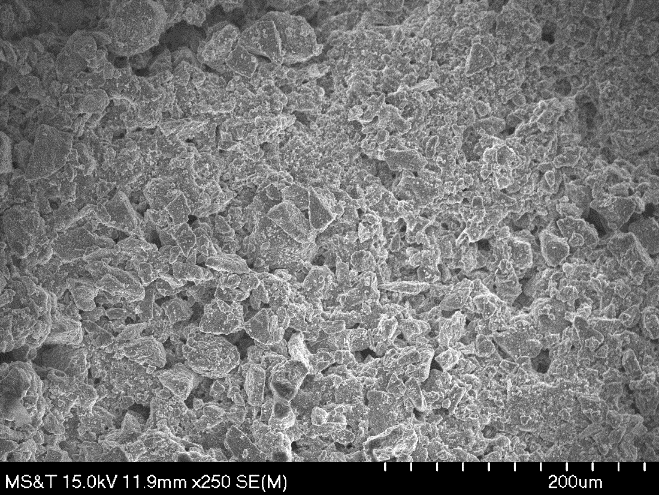

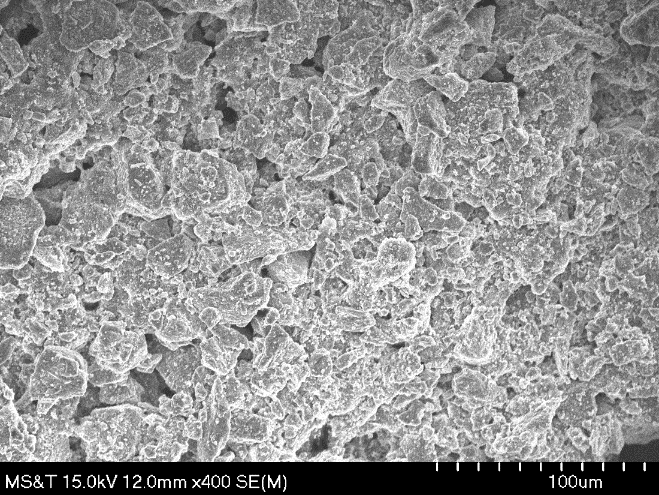

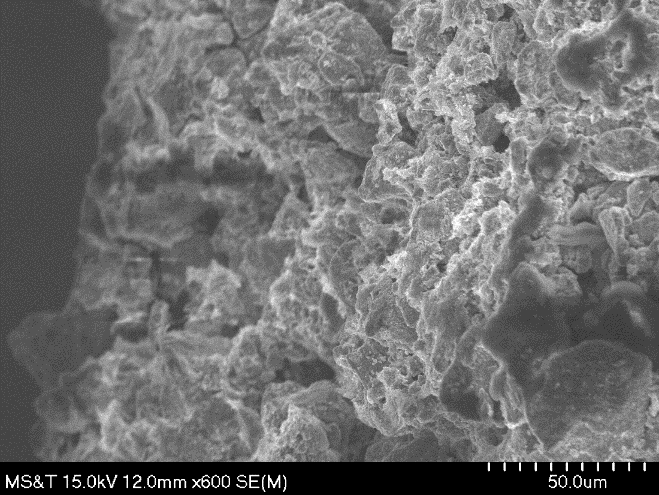

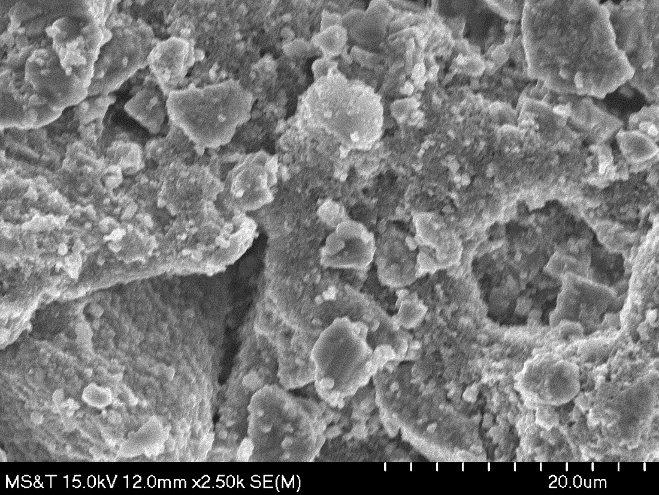


(a)

(b)

(c)

(d)

Figure S4. SEM images of electrodes without an electric field: (a) and (b) general view of the electrode, (c) edge of the electrode, and (d) zoomed view of the electrode.


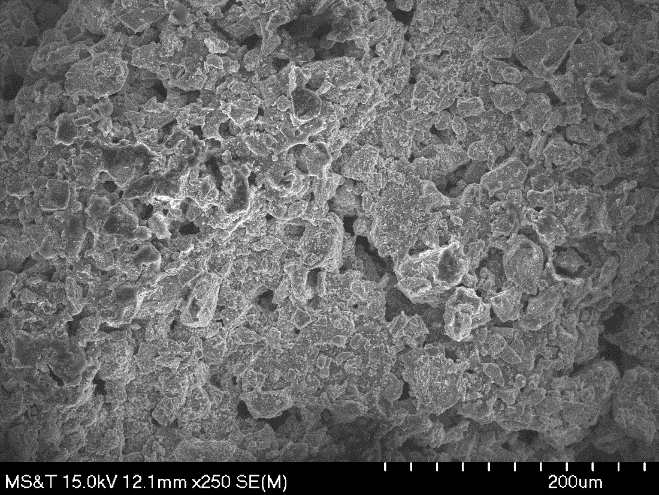

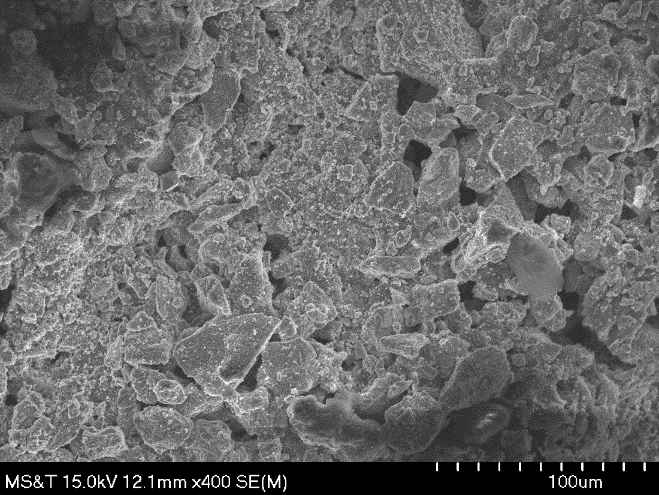

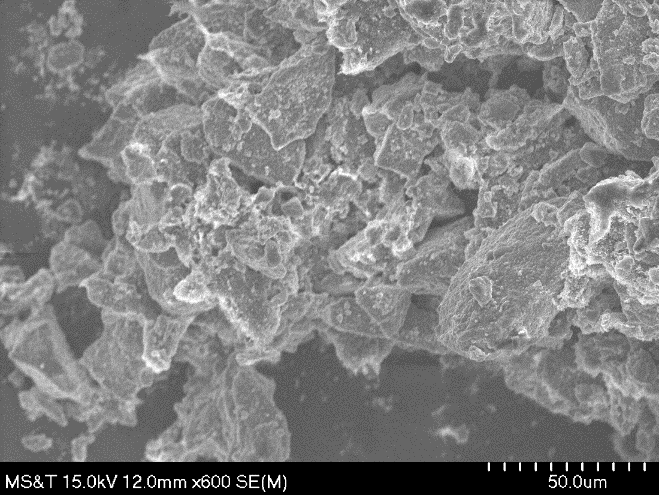

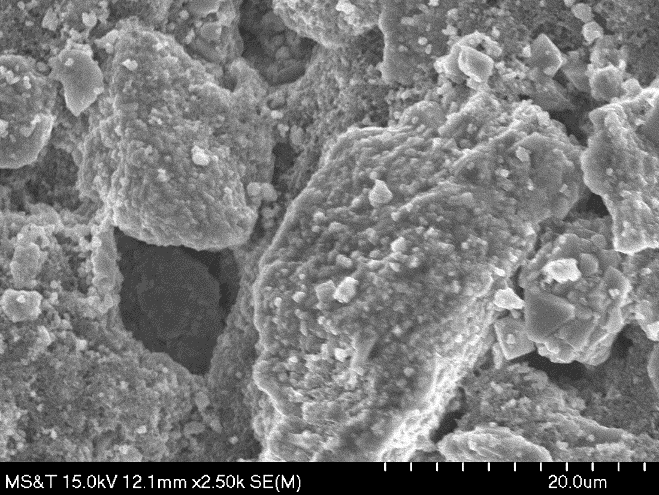


(a)

(b)

(c)

(d)

Figure S5. SEM images of electrodes with an electric field: (a) and (b) general view of the electrode, (c) edge of the electrode, and (d) zoomed view of the electrode.
